# Death of Mr. Tyrrell

**Published:** 1843-07-01

**Authors:** 


### DEATH OF MR. TYRRELL.

It is with much regret that we record the death of this eminent surgeon, which took place suddenly on the 23rd instant. The following were the appearances presented on a post-mortem examination.

*Chest.*—The pleura closely adherent throughout the whole of the right side of the chest, by old adhesive bands. On the left by a few only.

The right lung hepatized, or consolidated, nearly throughout its whole extent; there being only a very small portion that was crepitant on pressure. The mucous membrane of the lower portion of the trachea and right bronchus thickened and of a dark brick-dust hue. Left lung engorged with blood, but crepitating tolerably on pressure.

The pericardium considerably distended, and containing from an ounce and a half to two ounces of dark fluid-blood. On the posterior aspect of the right auricle the investing pericardium, to a considerable extent, had a rough, depressed, and by the aid of a lens, an ulcerated appearance; while the opposite surface of the loose pericardium, had a granular or tuberculated appearance, very similar to the tuberculous accretions which are found occasionally on the peritoneum; there were two or three adhesive bands connecting these two surfaces.

The heart itself was twice the natural size; the muscular fibre pale, flaccid, and flabby, and collapsing; the cavities of both ventricles much dilated, but without hypertrophy of the walls; the left ventricle more especially dilated; in the right ventricle were two or three delicate filaments of lymph of considerable strength, stretching between the carneæ columnæ and the chordæ tendiniæ, and across the cavity, the evident result of former endocarditis. All the valves of the heart and aorta were perfectly natural.

The liver was enlarged, very much indurated, especially at the superior portion of the right lobe, and granular throughout.

